# The potential utility of non-invasive imaging to monitor restoration of bladder structure and function following subtotal cystectomy (STC)

**DOI:** 10.1186/s12894-015-0094-6

**Published:** 2015-10-14

**Authors:** David Burmeister, Bimjhana Bishwokarma, Tamer AbouShwareb, John Olson, Maja Herco, Josh Tan, Karl-Erik Andersson, George Christ

**Affiliations:** Wake Forest Institute for Regenerative Medicine, 391 Technology Way, Winston-Salem, NC 27101 USA; Departments of Biomedical Engineering and Orthopaedic Surgery, and Laboratory of Regenerative Therapeutics, University of Virginia, 415 Lane Road, Charlottesville, VA 22908 USA; Wake Forest Department of Biomolecular Imaging, Medical Center Blvd, Winston-Salem, NC 27157 USA

**Keywords:** Subtotal cystectomy, Magnetic resonance imaging, Computed tomography, Regeneration, Urinary bladder

## Abstract

**Background:**

Restoration of normal bladder volume and function (i.e., bioequivalent bladder) are observed within 8 weeks of performing subtotal cystectomy (STC; removal of ~70 % of the bladder) in 12-week old rats. For analysis of bladder function in rodents, terminal urodynamic approaches are largely utilized. In the current study, we investigated the potential for Computed Tomography (CT) and Magnetic Resonance Imaging (MRI) scans to noninvasively track restoration of structure and function following STC.

**Methods:**

Twelve week old female Fisher F344 rats underwent STC and were scanned via CT and/or MRI 2, 4, 8, and 12 weeks post-STC, followed by urodynamic testing. After euthanasia, bladders were excised for histological processing.

**Results:**

MRI scans demonstrated an initial decline followed by a time-dependent increase to normal bladder wall thickness (BWT) by 8 weeks post-STC. Masson’s trichrome staining showed a lack of fibrosis post-STC, and also revealed that the percent of smooth muscle in the bladder wall at 2 and 4 weeks positively correlated with pre-operative baseline BWT. Moreover, increased BWT values before STC was predictive of improved bladder compliance at 2 and 4 weeks post-STC. Cystometric studies indicated that repeated MRI manipulation (i.e. bladder emptying) apparently had a negative impact on bladder capacity and compliance. A “window” of bladder volumes was identified 2 weeks post-STC via CT scanning that were commensurate with normal micturition pressures measured in the same animal 6 weeks later.

**Conclusions:**

Taken together, the data indicate some limitations of “non-invasive” imaging to provide insight into bladder regeneration. Specifically, mechanical manipulation of the bladder during MRI appears to negatively impact the regenerative process *per se*, which highlights the importance of terminal cystometric studies.

## Background

Tissue engineering and regenerative medicine technologies represent a promising approach for development of novel therapeutics for diverse lower urinary tract pathologies [1, 2]. In fact, many different animal models have been employed to evaluate the effectiveness of different cell/scaffold combinations in augmentation of the bladder [3–15]. However, a recent clinical report on bladder augmentation with autologous cell seeded biodegradable scaffolds clearly indicates that current technologies are not yet ready for widespread clinical applications [16]. Such developments speak to the importance of improved understanding of mechanisms of bladder regeneration, repair and remodeling *per se* as an important prerequisite to improved clinical applications of regenerative medicine/tissue engineering technologies to bladder dysfunction and disease.

Several studies have suggested that removal of a large part of the bladder without replacement results in some degree of functional bladder regeneration in both rats [17–22] and humans [23–32]. As such, we have focused our recent efforts on developing rodent models and methods that can provide additional insight into the cellular and molecular mechanisms responsible for functional bladder regeneration in mammals. In rodents, however, examining functional regeneration using urodynamic approaches is invasive and limited to evaluation of a single time point [33]. While voiding pattern assays provide an opportunity for longitudinal analysis of bladder function [34, 35], they are also limited in the amount of mechanistic physiological detail they can provide. As such, non-invasive imaging may permit longitudinal analysis of the bladder, providing additional morphological information over time, and potentially leading to novel insights on bladder functional restoration. The goal of this study was to evaluate the potential utility of non-invasive methods for improved understanding of the time course of functional bladder restoration following STC.

Furthermore, if successful, non-invasive imaging could also have great prognostic value in the clinic. Imaging techniques have already been applied to tissue engineering in, for example, cardiac, cartilage and bone regeneration [36–38]. Although a few studies have used Magnetic Resonance Imaging (MRI) and Computerized Tomography (CT) to evaluate the utility of grafts in the bladder, little is known about how changes in the morphology of the bladder relate to bladder function [39–41]. The current study explores the utility of both CT and MRI to inform understanding of bladder function during regrowth/regeneration induced by subtotal cystectomy (STC) in a well-characterized rodent model. The results of this study demonstrate a potential for non- invasive imaging as a prognostic indicator for the functional success or failure of bladder regeneration.

## Methods

### Animals

**Fig. 1:**
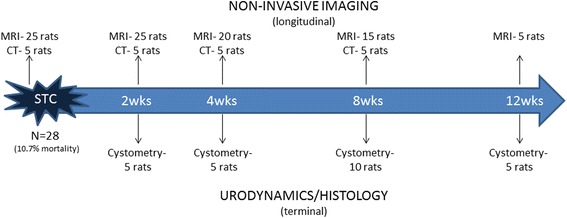
Schematic showing experimental design. Of the 28 rats undergoing STC, 3 died post-operatively, and the baseline scans of those animals were omitted. All rats underwent MRI at every timepoint, while 5 of those rats had CT scans performed at every timepoint until 8 weeks. 5 rats were used for terminal urodynamic studies at 2, 4, and 12 weeks. 10 rats had urodynamic testing at 8 weeks, including the 5 rats that received CT scans. STC- subtotal cystectomy

Twenty-eight 12-week old (170–200 g) female Fisher F344 rats underwent subtotal cystectomy (STC), and the experimental design is shown in Fig. 1. All animals underwent MRI scanning pre-operatively, with cohorts of animals also scanned at 2, 4, 8, and 12 weeks post-STC. For cystometric studies, 5 animals were designated for analysis at 2, 4, and 12 weeks, while 10 were designated for analysis at 8 weeks. Of these 10 animals at 8 weeks post-STC, half (*n* = 5) were utilized for CT scanning in addition to MRI. MRI procedures were completed the day before CT scanning and catheter implantation. Additionally, retrospective analysis was performed on previously published data from animals of the same strain, age, and gender [21]. All methods were approved by the Animal Care and Use Committee, Wake Forest University.

### Trigone-sparing cystectomies

Animals underwent trigone-sparing STC as previously described [21, 22, 42]. Briefly, two stay sutures were made on either side of the bladder, just above the uretero-vesical junction, using 6–0 polyglycolic acid. The dome portion of the bladder was excised and the remaining portion of the bladder was then sutured continuously using one of the original stay sutures. Animals were allowed to recover and given food and water *ad libitum*.

### Magnetic resonance imaging (MRI)

All MRI experiments were performed in a 7 T horizontal bore magnet (Bruker Biospin, Billerica, MA) equipped with an actively shielded gradient insert. RF signal transmission and reception was performed with a 50 mm I.D. quadrature Litzcage RF coil (Doty Scientific, Columbia, SC) tuned and matched with each rat to 300.2 MHz. Rats were anesthetized with oxygen (2 L/min) and isoflurane (2 %) and the bladder was manually expressed. The animal was then placed in the RF coil in the prone position with the bladder centered in the RF coil. Anesthesia was maintained during the scan via nose cone which provided oxygen (1 L/min) and isoflurane (1.5 %). Body temperature was kept constant by thermostatically controlled warm air (SA Instruments, Stoney Brook, NY). A three plane localizer scan was acquired using a Rapid Acquisition with Relaxation Enhancement (RARE) spin echo pulse sequence with an echo train of 8 echos to ensure that the bladder of each rat was centered in both the RF coil and the magnet. A 3D FLASH pulse sequence allowed for the acquisition of 8 slices, each 250 microns thick. The low slice thickness reduced partial volume effects, thus allowing for better detection of the inner and outer surfaces of the bladder wall. The coronal 3D FLASH slab was positioned using the tri plane localizers as scout images so that the slices in the center of the slab were perpendicular to the surface of the bladder wall. Scanning parameters were as follows: TR = 50 ms, TE = 6 ms, flip angle = 15 degrees, FOV = 3 cm, matrix = 256x256, giving an in-plane resolution of 117um, NEX = 8. Acquisition was also respiratory gated to avoid breathing motion artifacts. Analysis of bladder wall thickness was performed using TeraRecon 3D visualization and image analysis software using the linear measurement tool. This was performed in triplicate, on five different locations of the bladder wall, denoted “base” to “dome”.

### *CT imagin*g

The day after MRI testing, CT Scans of the animals were taken with a Siemens MicroCATII @ 70 kV, 500 μA (BIN Factor of 4, 200° rotation, 500 steps, 73 micron cuts), and the scans were centered on the bladder. Contrast medium (288 mg/ml Iothalamate Meglumine, diluted 1:3) was applied via transurethral catheterization and injected until bladder was full. All images were reconstructed using COBRA EXXIM version 4.9.52, and converted to DICOM images with Amira version 3.1. Analysis was done after transfer of images to TeraRecon Aquarius Workstation. Briefly, the entire image except the bladder was removed using the erase function. A bladder template was optimized with the following parameters: Window Width (WW) of 1116, Window Level (WL) of 657, and 11 % opacity. Finally, the volume measurement tool was used for quantification of bladder volume.

### Cystometric analysis

Bladder catheters were implanted and cystometric studies were performed 3 days after catheter implantation in conscious, freely moving rats as previously described [21, 42, 43]. Briefly, the indwelling catheter was connected to a pressure transducer and infusion pump. The pressure transducer was connected to an ETH 400 (CD Sciences, Dover, New Hampshire) amplifier and read with a MacLab/8e (Analog Digital Instruments, New South Wales, Australia) acquisition board. Equipment was calibrated in cmH_2_0 before each experiment. Room temperature saline was infused at a rate of 10 mL/h. Voided fluid was diverted into a collection tube attached to a force displacement transducer. The following cystometric parameters were investigated: basal pressure (BP, lowest pressure between voids), maximum pressure (MP, the highest pressure during micturition), threshold pressure (TP, pressure which initiates a voiding contraction), bladder capacity (B_cap_, residual volume plus amount of saline infused), micturition volume (MV, amount of expelled urine), residual volume (RV, B_cap_ –MV), and bladder compliance (B_com_ = B_cap_/(TP-BP)).

### Histology

An additional subset of bladders were preserved for histological analysis of smooth muscle content (*n* = 4/timepoint). Bladders distal to the UVJ were fixed in 10 % buffered formalin overnight, processed, embedded in paraffin and then cut into 7 μM axial slices. Slides were cleared in xylene and rehydrated to water. Masson’s trichrome stain (Newcomer Supply Catalog #9176A) was performed on at least 2 different areas of the bladder (i.e. a section closest to the base or UVJ/original plane of excision, and a section taken towards the dome or more distally). Four high magnification images were taken in each section, and image analysis was performed with ImagePro software 6.3 (Media Cybernetics, Bethesda, MD). The color selection tool was used to determine quantity of red (muscle) and blue (collagen) pixels, and the percentage of muscle corresponds to the number of red pixels/total number of selected pixels.

### Statistical analysis

Non-invasive image reconstruction was performed with TeraRecon AquariusNET software version 4.4.5.49 (TeraRecon, Inc., San Mateo, CA). Statistical evaluations and regression analysis were performed using GraphPad Prism software. (GraphPad software Inc.) One-way ANOVAs with Neumann-Keuls post testing were performed on bladder wall thickness, cystometric parameters, and Trichrome analysis. Additionally, t-tests were performed on cystometric parameters obtained at 8 weeks in order to determine the effect of MRI. A two-way ANOVA was used to determine any regional variations in bladder wall thickness. P values less that 0.05 were considered significant. All results are expressed as the mean ± SEM.

## Results

### Subtotal cystectomy

Of the 28 rats that underwent STC, three animals died within three days after surgery due to urine leakage into the peritoneum (10.7 % mortality rate).

### MRI scanning

**Fig. 2:**
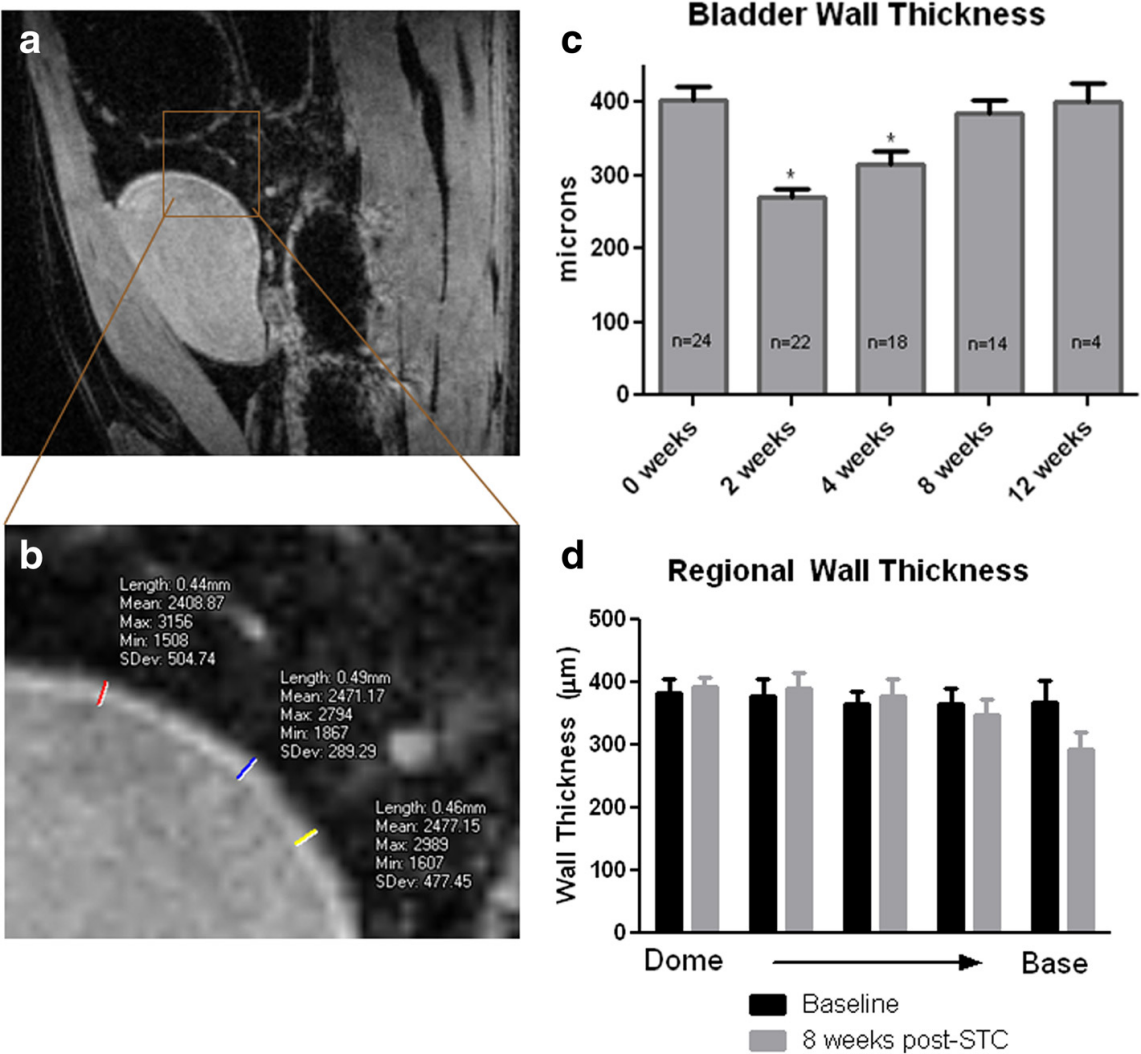
Bladder wall thickness normalizes thickness 8 weeks after STC. **a** Example of a sagittal view of a control (pre-STC) bladder visualized by magnetic resonance imaging (MRI), magnified in **b**. **c** 1 Way ANOVA analysis of quantified bladder wall thickness using MRI scans reveals that the bladder wall is thinner than pre-STC values 2 and 4 weeks post-STC (*P* < 0.01). The number of observations at each timepoint represent the number of successful scans at each timepoint, as instances of gating artifact did lead to some unsuccessful scans. The ratio of unsuccessful scans to total number of possible scans was 1/25, 3/25, 2/20, 1/15, and 1/5 at 0, 2, 4, 8, and 12 weeks, respectively. **d** Analysis of regional wall thickness shows no differences from control (*n* = 24) and 8 weeks post-STC (*n* = 14)

An example of a sagittal bladder slice and associated measurements are shown in Fig. 2a, with demonstration of measurements in Fig. 2b. Analyzable scans were attained in 24/25, 22/25. 18/20, 14/15, and 4/5 possible scans at the 0, 2, 4, 8, and 12 week time points, respectively. Analysis of sagittal slices of MRI scans revealed an initial decrease in bladder wall thickness (BWT) after STC, which normalized to control values by 8 weeks post-STC (Fig. 2c). Values for BWT were 402.1 ± 18.82 microns, 269.6 ± 12.21 microns, 315.3 ± 17.55 microns, 384.7 ± 19.11 microns, and 399.8 ± 25.12 microns at the 0, 2, 4, 8, and 12 week time points, respectively. Analysis of BWT variations from the bladder base to the bladder dome revealed no significant differences between any of the time points studied (8 weeks shown in Fig. 1d).

### Trichrome analysis and regression

**Fig. 3:**
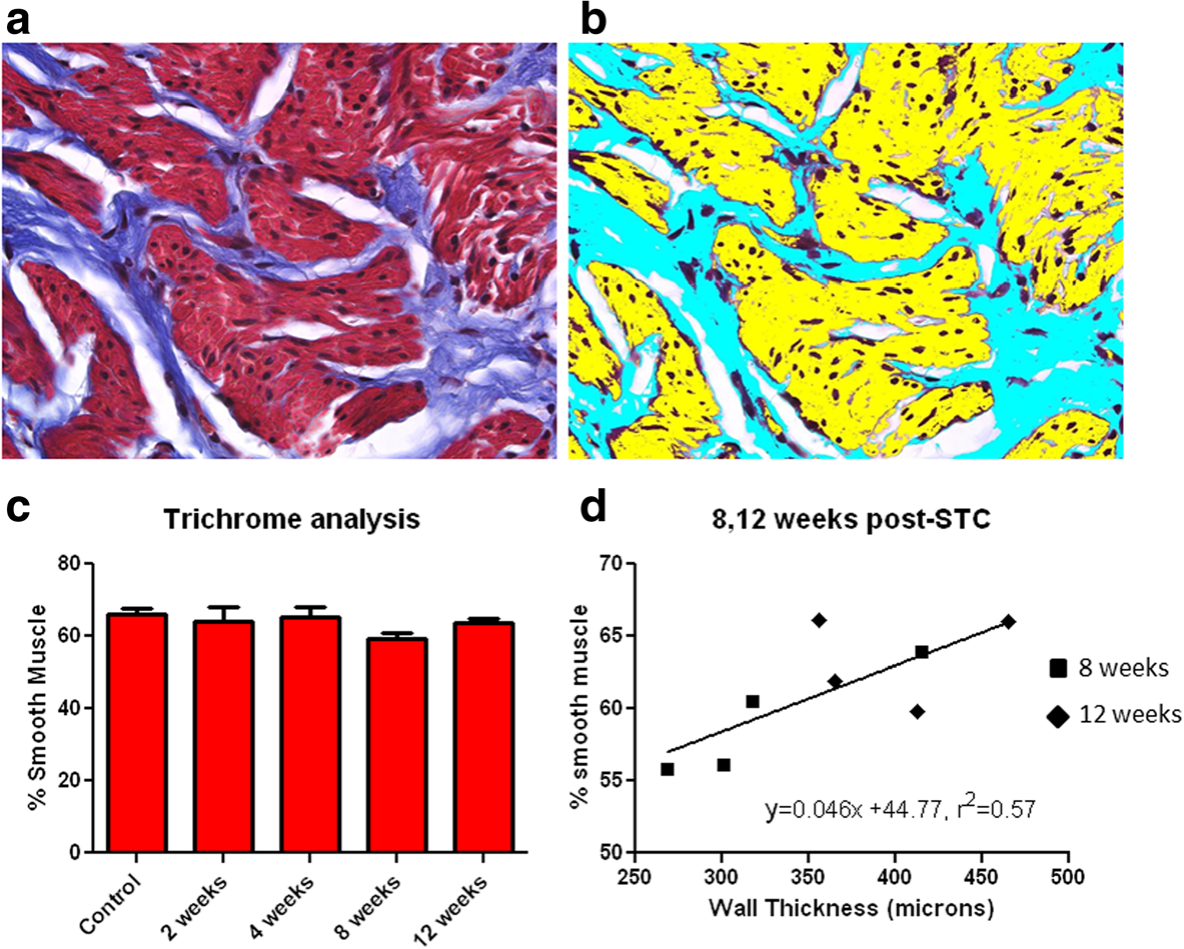
Quantification of smooth muscle to collagen ratios using semi-quantitation of Masson’s trichrome staining. **a** Representative image of excised STC bladder used for quantification by choosing pixel intensity **b. c** Analysis shows no differences accross time, however linear regression **d** reveals that the amount of smooth muscle is correlated with bladder wall thickness determined via MRI (*P* < 0.05)

A representative trichrome image is shown in Fig. 3 and analysis revealed no changes in percent smooth muscle values at any time after STC, which were 66.16 ± 1.41, 64.14 ± 3.35, 65.46 ± 2.09, 59.10 ± 1.67, and 63.48 ± 1.35 % at 0,2,4,8, and 12 weeks post-STC, respectively. However, the percent smooth muscle in the bladder wall at the 8 and 12-week time points was positively correlated with MRI-determined bladder wall thickness at those same time points.

### Cystometric analysis

**Fig. 4:**
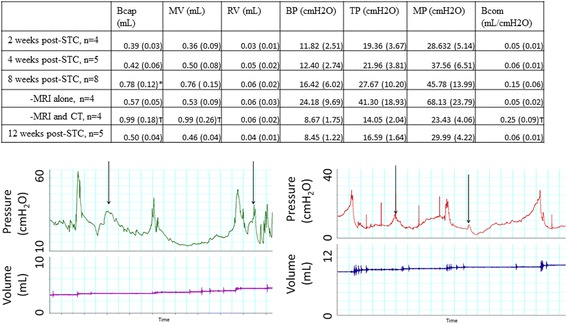
Urodynamic parameters as determined by in vivo cystometry. Top table shows mean (SEM) for animals 2, 4, 8, and 12 weeks post-STC. *- Bladder capacity was higher 8 weeks post-STC compared with all other timepoints (*P* < 0.05). Cystometric parameters at 8 weeks post-STC are further broken down into animals that were imaged via CT, and those that only underwent MRI scanning. Ϯ- Bcap, MV, and Bcom are significantly different between animals with MRI alone (*P* < 0.05). Bcap- Bladder Capacity, MV- Micturition Volume, RV- Residual Volume, BP- Basal Pressure, TP- Threshold Pressure, MP- Maximal Pressure. Bcom- Bladder Compliance. Lower panels show representative cystometrograms from animals at 8 weeks subjected to MRI alone revealing abnormal readings in the form of non-voiding contractions (arrows)

All cystometric parameters are displayed in Fig. 4. Bladder capacity was higher at 8 weeks compared to every other time point post-STC. Consistent with previous studies, the average measured bladder capacity increased until the 8-week time point, however in contrast to those studies, bladder capacity declined afterwards. Upon closer inspection, there was significant variation in bladder capacity at the 8-week time point (the only time point at which CT was also performed). Subdivision of the animals at the 8-week time point into those animals that received both CT and MRI vs. those that received MRI alone revealed statistically significant increases in bladder capacity, micturition volume and bladder compliance in CT-scanned animals when compared to animals that had MRI alone. Importantly, bladders still emptied completely in all animals as evidenced by low residual volume.

### Linear regression of urodynamic parameters and MRI Scans

**Fig. 5:**
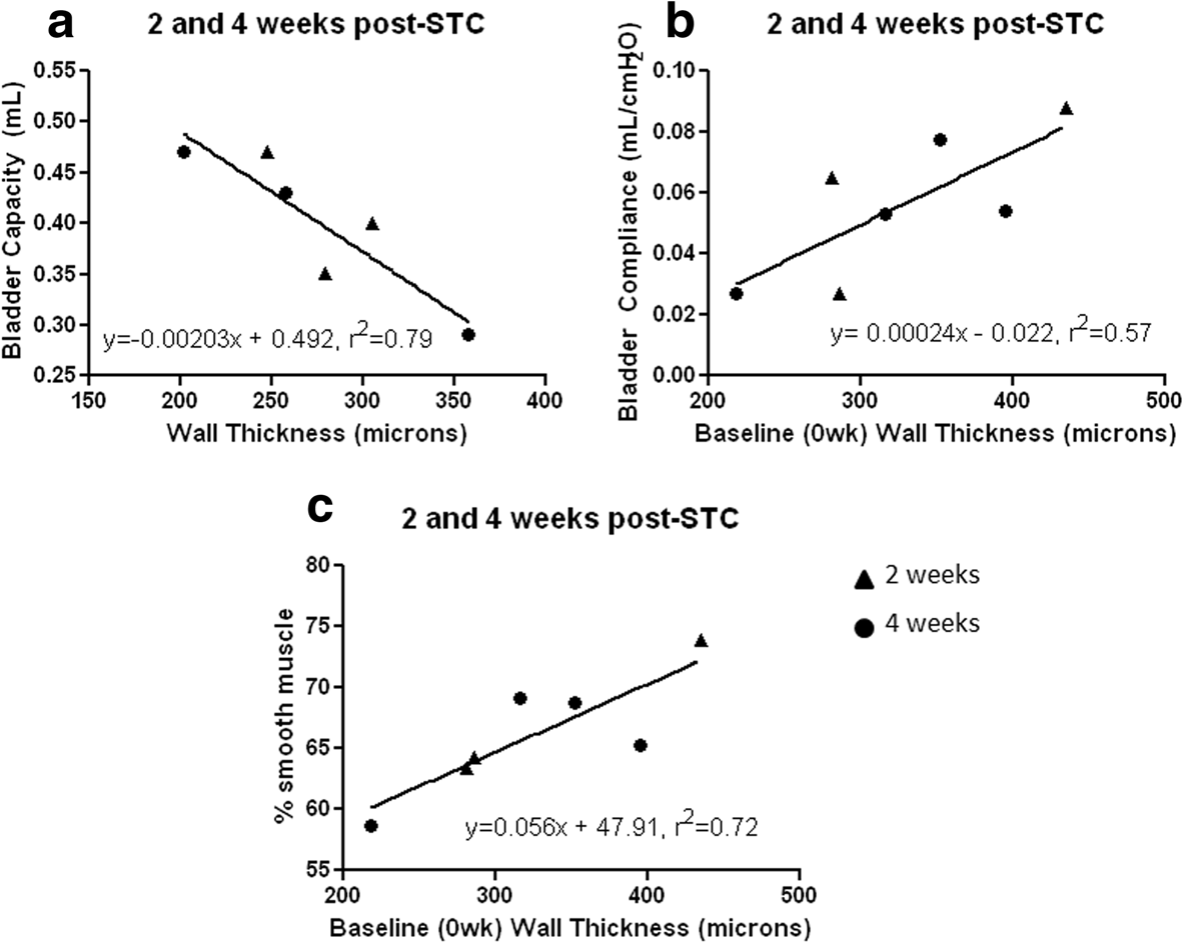
Linear regression analysis of cystometric parameters with MRI-determined bladder wall thickness. **a** At early timepoints (i.e., 2,4 weeks) after STC, the bladder capacity is negatively correlated with bladder wall thickness at that same timepoint. The pre-operative thickness of the bladder wall (baseline) is positively correlated with bladder compliance (**b**) and smooth muscle content (**c**) seen at early time points post-STC. (*P* < 0.05)

Linear regression analysis revealed that BWT, as determined via MRI (see Fig. 5), correlated with some cystometric parameters early post-STC (i.e., 2 and 4 weeks). Specifically, BWT at the time of sacrifice was negatively correlated with bladder capacity at that same time point (Fig. 5a). Additionally, pre-operative (baseline) BWT values were positively correlated with the bladder compliance and percent smooth muscle seen at 2 and 4 weeks post-STC (*P* = 0.049, and 0.016, respectively).

### CT scanning

**Fig. 6:**
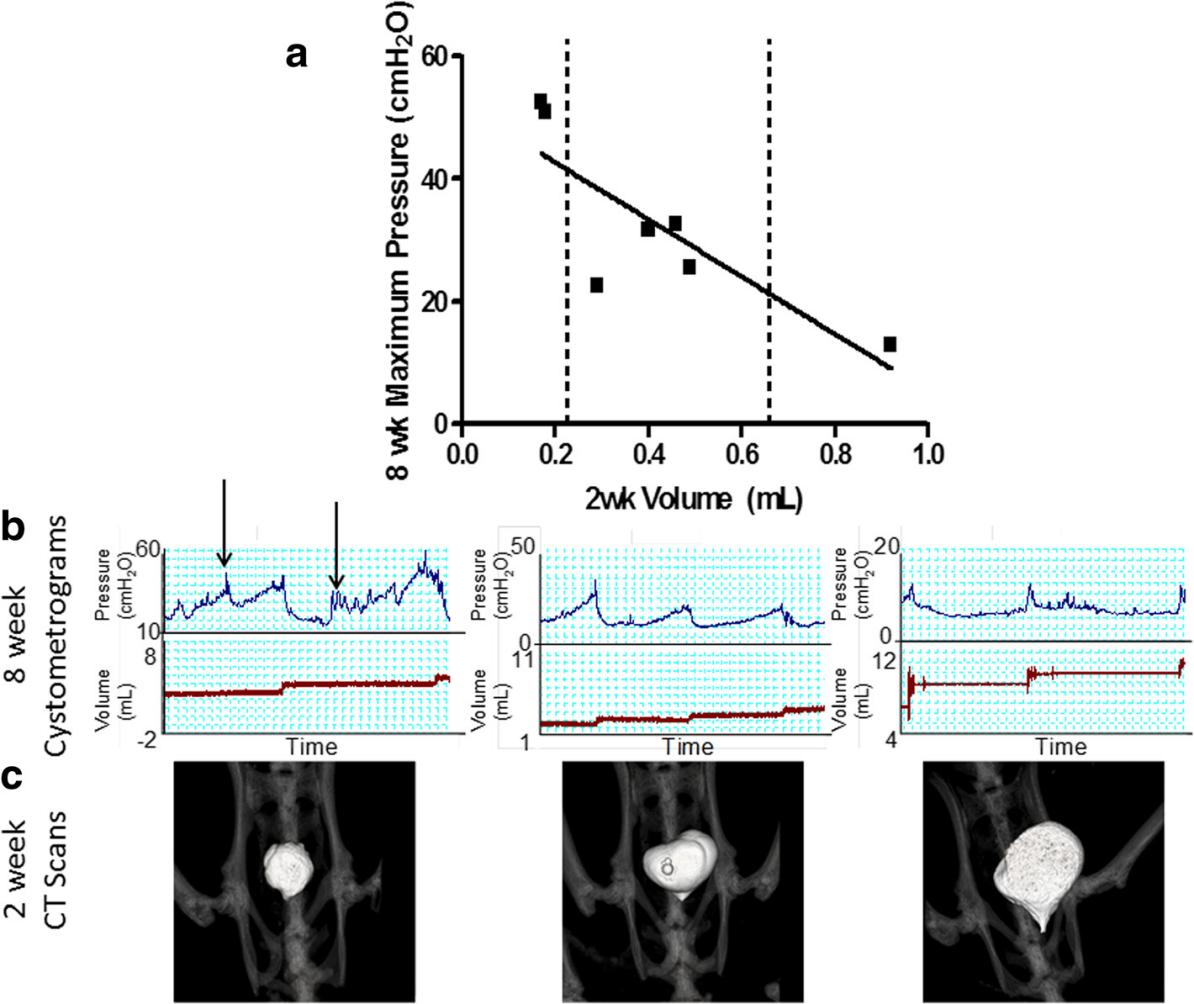
Retrospective regression analysis of animals subjected to STC reveals a predictive value for CT Scanning. This graph represents 2 week CT and 8 week cystometric data from 4 animals in this study, along with 3 animals from a previous study (Burmeister et al., 2010). **a** X-axis represents bladder volume 2 weeks post-STC as determined by CT Scanning, and Y-axis represents maximum pressure (MP) generated by bladders 8 weeks post-STC as determined by *in vivo* urodynamic studies in the SAME ANIMAL. **b** Representative cystometrograms at 8 weeks and (**c**) CT scans at 2 weeks of 3 individual animals. This analysis displays that animals with minimal bladder growth seen upon CT scanning 2 weeks post-STC (left of the dashed lines) display bladder overactivity (arrows show non-voiding contractions), while the animal with a large bladder volume 2 weeks post-STC (right of the dashed lines) generates low maximum pressures (i.e. ~13 cmH_2_O). Normal bladder function was seen in animals with intermediate bladder volumes determined by CT 2 weeks post-STC. *y* = −46.65x + 52.12, *r* = 0.82, *P* = 0.02

A previous report documented that the anterior bladder circumference measured via CT scans positively correlated with maximum pressures generated by the bladder post-STC [21]. Here, we conducted a retrospective analysis to further examine CT-determined bladder volume with other cystometric parameters from the same animal. Retrospective analysis revealed a negative correlation between total bladder volumes measured by CT scans 2 weeks post-STC and the maximum pressures generated *in vivo* 8 weeks post-STC (Fig. 6). A bladder volume of less than 0.2 mls at 2 weeks post-STC was associated with high pressures and detrusor overactivity at 8 weeks (i.e., the appearance of non-voiding contractions). Conversely, a bladder with a large volume at 2 weeks (>0.8 mls) resulted in significantly diminished pressure generation during micturition at 8 weeks post-STC. Bladders in which volumes ranged between 0.2 and 0.6 mls 2 weeks post-STC estimated by CT imaging were subsequently observed to have normal urodynamic profiles at 8 weeks post-STC.

## Discussion

The present study investigated the utility of Magnetic Resonance Imaging (MRI) and Computerized Tomography (CT), in conjunction with traditional measures, to provide longitudinal mechanistic insight into restoration of bladder structure and function. MRI can be challenging in rodent models because of the small size of organs/tissues (rat bladder). We were able to overcome difficulties by using a 7 Tesla magnet coupled with respiratory gating, although a few instances of motion artifact prevented acquisition of analyzable images (8/90 possible scans). We noted that bladder wall thickness (BWT) drastically decreased immediately after STC, but returned to normal pre-operative values by 8 weeks post-STC (Fig. 2). Although absolute values of BWT reported here with MRI (≈400 μm) are lower than our previous report based on histological evaluation (≈550 μm), it is still within the normal range reported for BWT [21, 44]. Discrepancies between tissue thickness via MRI and histology have been shown previously in, for example, the retina [45, 46]. Although statistically thinner at 2–4 weeks after STC, the bladder did not display any significant fibrosis at any time point, as illustrated by the percent smooth muscle in the bladder wall displayed in Fig. 3c.

Urodynamic studies revealed increased bladder capacities 8 weeks post-STC similar to our prior report [21]; however this was not maintained 12 weeks post-STC (Fig. 4). Significant variability in cystometric parameters 8 weeks post-STC prompted subdivision of the animals based on whether they had an MRI only or MRI plus CT on the day following MRI. Manual bladder emptying during MRI apparently had an adverse impact on the regeneration process leading to instances of non-voiding contractions, abnormal basal pressures, and small bladder capacities (Fig. 4). At all time points, animals assessed via MRI showed diminished recovery of normal bladder volumes. Conversely, animals in which MRI scanning/bladder emptying was followed by bladder filling during CT scanning showed complete restoration of bladder volume and improved bladder compliance. While the precise mechanism(s) responsible for this observation remain unclear, it is interesting to speculate that it may be related to untoward mechanical manipulation of the bladder emptying during critical periods of bladder regrowth and regeneration. Consistent with this supposition is recent data in a murine model of STC (Christ et al., unpublished observations) that indicates that MRI without bladder emptying results in normal recovery of bladder volume. Despite diminished recovery of bladder volume in this study, residual volume never increased in any animal, and there was no decrease in bladder wall thickness (Fig. 2) or smooth muscle percentage in the bladder wall (Fig. 3).

A limitation of the current study involves technical difficulties in standardizing the state of the bladder during imaging (i.e. how full/empty the bladder is during scanning). Ideally, the bladder would be filled to a standard pressure during scans to atone for dynamic BWT due to filling. However, these MRI scans lasted approximately one hour, and starting from empty presumably minimizes the effect on BWT due to physiological filling. Similarly, CT scanning requires filling the bladder with contrast medium, and clamping of the catheter to prevent emptying. It is logistically difficult to ensure a consistent pressure during these scans, resulting in comparison of thoroughly and comparably full bladders. Regardless, there is reasonable consistency in these procedures as reflected in small variability shown in MRI and CT parameters. While we acknowledge these technical limitations, as noted above, the BWT measurements derived from MRI were still a reasonable approximation of prior reports using more standard measures [21, 44].

Given these considerations, MRI studies still yielded potentially interesting relationships between BWT and bladder capacity and compliance (Fig. 5). Specifically, during the first month of bladder regeneration, BWT was negatively correlated with bladder capacity, perhaps due to non-optimal cellular proliferation/organization in the regenerating bladder wall at early time points. We also found that baseline BWT (i.e., pre-STC) was positively correlated with both bladder compliance and the percent of smooth muscle found in the bladder during the first month post-STC. These correlations point to an overall improved outcome of bladder regeneration in animals that have a thicker bladder wall pre-STC, and earlier recovery of BWT post-STC. While these metrics, as well as their putative mechanism(s) require further investigation, they may provide valuable insight into important correlates of successful regenerative responses in the bladder.

A previous report documented that bladder volumes estimated by CT imaging accurately tracked bladder capacity measured via cystometry [21]. Here we report a retrospective analysis of those results, illustrating a significant correlation observed between CT-determined bladder volumes 2 weeks post-STC and maximal pressure determined cystometrically in the same animal 8 weeks post-STC. This suggests that there may be a normal volume range for bladder re-growth during the first two weeks post-STC, such that bladder volumes outside this range may result in abnormal bladder function (Fig. 6b). Specifically in the 2 weeks post-STC, a small increase in bladder capacity (<0.2 mls) results in bladder overactivity (i.e. high pressure and non-voiding contractions), while a large amount of bladder growth (>0.8) results in a substantial deficiency for pressure generation *in vivo*. If further validated as an index for the eventual success of bladder regeneration, the use of CT scans at early time points during bladder regrowth may provide a critical opportunity for intervention(s) to correct an otherwise failed therapeutic recovery.

## Conclusions

To summarize, we have demonstrated that although non-invasive imaging may be a useful tool for obtaining mechanistic insight into bladder regeneration during the first 3 months post-STC, these methods alone are not yet ready to replace well-established functional analyses (i.e. cystometry), as well as more descriptive measures of bladder voiding patterns [35, 36]. Specifically, the impact of mechanical emptying of the bladder during critical stages of regeneration using these modalities must be carefully monitored to ensure that their utilization does not affect the remodeling response being measured. Further investigation will hopefully identify appropriate boundary conditions such that both CT and MRI can be more effectively used and provide important noninvasive mechanistic insight into functional bladder regeneration. While current urodynamic approaches (i.e. cystometry and voiding pattern analysis) must still be employed, non-invasive imaging may eventually allow researchers to follow important aspects of the morphogenesis/bladder regeneration longitudinally. Ultimately, this approach could identify noninvasive metrics early on in the regenerative process where one might be able to not only predict the extent and outcome of regeneration/re-growth, but also develop effective interventions for therapeutic restoration of bladder re-growth/regeneration.

## Abbreviations

STC: Subtotal Cystectomy
BWT: Bladder Wall Thickness
MRI: Magnetic Resonance Imaging
CT: Computed Tomography
